# Salicylic Acid Mitigates Drought-Stress-Induced Oxidative Damage in *Ilex rotunda* Through Tissue-Specific Reprogramming of Antioxidant Phenolics and ROS Scavenging

**DOI:** 10.3390/antiox15070808

**Published:** 2026-06-27

**Authors:** Huwei Yuan, Qinyuan Shen, Ye Zheng, Mingzheng Duan, Junhan Guo, Yihui Li, Jiashuang Qiao, Liangye Huang, Maryam Tahira, Yanyan Yin, Jiaxin Hu, Jianfang Zuo, Daoliang Yan, Bingsong Zheng, Muhammad Junaid Rao

**Affiliations:** 1 State Key Laboratory for Development and Utilization of Forest Food Resources, Zhejiang A&F University, Hangzhou 311300, China; hwyuan@zafu.edu.cn (H.Y.); 2024202011016@stu.zafu.edu.cn (Q.S.); 2024102012044@stu.zafu.edu.cn (Y.Z.); guojh@stu.zafu.edu.cn (J.G.); 2024602122054@stu.zafu.edu.cn (Y.L.); 2025602122079@stu.zafu.edu.cn (J.Q.); 2023602121036@stu.zafu.edu.cn (L.H.); yinyan@stu.zafu.edu.cn (Y.Y.); hujiaxin123@stu.zafu.edu.cn (J.H.); zuojianfang@zafu.edu.cn (J.Z.); liangsie@zafu.edu.cn (D.Y.); bszheng@zafu.edu.cn (B.Z.); 2 Advanced Institute of Ecological Agriculture and Biodiversity on the Yunnan-Guizhou Plateau/Yunnan Key Laboratory of Smart Villages and Agri- Cultural- Tourism Integration, Zhaotong University, Zhaotong 657000, China; 3 Zhejiang Key Laboratory of Non-Wood Forest Products Quality Regulation and Processing Utilization, Zhejiang A&F University, Hangzhou 311300, China; 4 National Key Laboratory for Germplasm Innovation & Utilization of Horticultural Crops, College of Horticulture and Forestry, Huazhong Agricultural University, Wuhan 430070, China; maryam@webmail.hzau.edu.cn

**Keywords:** *Ilex rotunda*, drought resilience, UPLC-MS/MS, caffeoylquinic acids, tissue-specific metabolism, medicinal woody perennials, antioxidants

## Abstract

Drought stress imposes oxidative damage on plants, yet the tissue-specific roles of salicylic acid (SA) in modulating phenolic metabolism remain poorly understood in woody species. Using *Ilex rotunda* seedlings, we investigated whether exogenous SA (100 µM) mitigates drought-induced oxidative damage and reshapes phenolic profiles in different tissues. Drought alone increased leaf total phenolics by 32% but depleted root phenolics by 29%, whereas combined drought + SA (DSA) treatment partially restored root phenolic levels, coinciding with elevated malondialdehyde (MDA) (2.2-fold in leaves, 2.6-fold in roots) and H_2_O_2_. Leaf antioxidant capacity increased under drought (DPPH by 73%, •OH by 33%), whereas root DPPH declined by 27% despite a 26% rise in •OH scavenging. SA alone induced mild oxidative responses and selectively upregulated caffeoylquinic and galloyl derivatives, notably 1-Caffeoylquinic acid (log_2_FC = 6.38) in leaves. DSA treatment mitigated oxidative damage—reducing leaf MDA by 44% and root H_2_O_2_ by 38%. Metabolomics revealed tissue-specific reprogramming leaves accumulated dicaffeoylshikimic acid (log_2_FC = 10.66) and trilobatin D (log_2_FC = 11.18) under DSA, whereas roots showed contrasting patterns with up-accumulation of vanillate (log_2_FC = 5.77) and suppression of 3,5-dicaffeoylquinic acid (log_2_FC = −7.21) under drought, with stronger metabolic reprogramming in leaves than roots. Our findings indicate that SA-mediated drought tolerance is associated with tissue-specific phenolic reprogramming, identifying candidate indicators that advance the mechanistic understanding of woody plant resilience to drought. These results provide a framework for translating metabolomic signatures into practical strategies for stress mitigation in medicinal perennials facing climate change.

## 1. Introduction

Drought is one of the most pervasive abiotic constraints limiting plant growth, productivity and geographical distribution, and its frequency and intensity are projected to increase under ongoing climate change [1,2]. Water deficit disrupts cellular homeostasis, impairs photosynthesis and carbon assimilation, and ultimately constrains biomass accumulation and yield in both crops and woody perennials [3,4,5]. A central consequence of drought is the overproduction of reactive oxygen species (ROS), including superoxide anion (O_2_•^−^), hydrogen peroxide (H_2_O_2_) and hydroxyl radicals (•OH), in chloroplasts, mitochondria, peroxisomes and the apoplast [6,7,8]. When ROS generation exceeds the capacity of antioxidant systems, oxidative stress ensues, leading to lipid peroxidation, protein oxidation, nucleic acid damage and loss of membrane integrity [8,9,10]. Osmotic adjustment via compatible solutes (e.g., proline, soluble sugars) is a primary drought defense that stabilizes cellular hydration and protects macromolecules. Although this study focuses on phenolic antioxidants, these osmolyte and phenolic pathways are likely to operate cooperatively to confer whole-plant drought tolerance.

Plants have evolved a complex antioxidant network comprising enzymatic components, such as superoxide dismutase, catalase and peroxidases, and a diverse array of low-molecular-weight metabolites. Among these, phenolic compounds constitute a major class of non-enzymatic antioxidants with high radical-scavenging capacity and metal-chelating properties [11]. Phenolics encompass simple phenolic acids, flavonoids, tannins and a wide range of caffeoyl- and galloyl-derived conjugates that can directly quench ROS, stabilize membranes and modulate redox signaling [6,12]. Numerous studies have shown that drought and other abiotic stresses alter phenolic biosynthesis and accumulation, often enhancing total phenolic content and antioxidant capacity in leaves [8,13,14]. However, phenolic metabolism is highly compartmentalized at the tissue and organ levels. Roots and leaves can differ markedly in both the composition and abundance of phenolic subclasses, reflecting their distinct developmental programs and exposure to environmental cues [15,16,17,18,19,20].

Salicylic acid (SA) is a key phenolic phytohormone that regulates plant growth, immunity and abiotic stress responses [21]. Beyond its established role in systemic acquired resistance, SA modulates stomatal behavior, photosynthetic performance and antioxidant defenses under drought, salinity and temperature extremes [22,23,24]. Nevertheless, SA itself is structurally related to many specialized phenolics, and accumulating evidence suggests that it can reprogram phenylpropanoid and flavonoid biosynthesis [25]. How SA orchestrates the deployment of phenolic antioxidants across different tissues under water deficit is still poorly understood in *Ilex* species.

This knowledge gap is particularly critical for long-lived woody plants such as *Ilex rotunda* Thunb. (Aquifoliaceae), an evergreen medicinal tree widely distributed in East Asia [26]. Roots constitute the primary site of water deficit perception and mediate long-distance stress signaling to shoots. Root phenolic metabolism regulates hydraulic conductance and influences rhizosphere chemistry, thereby playing a critical role in whole-plant drought resilience. Although the bark is the primary medicinal organ [26], we focus on leaves and roots because drought stress is initially perceived and physiologically mitigated in photosynthetic and water-uptake tissues. Furthermore, destructive bark harvesting is unsustainable; understanding stress responses in non-medicinal organs can inform cultivation practices that preserve whole-plant health without sacrificing the tree. The dried bark of *I. rotunda*, known as “Jiu Bi Ying” (救必应) in traditional Chinese medicine, is officially listed in the Chinese Pharmacopoeia and has been used for centuries to treat inflammation, gastroenteritis, and rheumatic conditions [26,27]. Modern phytochemical studies have identified over 120 compounds from this species, with phenolic derivatives—including caffeoylquinic acids and phenolic glycosides, contributing to its notable antioxidant, anti-inflammatory, and cardioprotective properties [26,28,29]. Beyond their medicinal applications, phenolic compounds such as caffeoylquinic acids and galloyl derivatives are increasingly recognized as valuable natural antioxidants in food science and sustainable packaging. Recent studies have demonstrated that starch-based films and coatings enriched with plant-derived phenolics significantly enhance food shelf life by reducing lipid oxidation and inhibiting microbial growth [30]. Similarly, edible coatings infused with natural antioxidants emerging as viable alternatives to synthetic plastic packaging, offering both preservation benefits and environmental sustainability [31]. The tissue-specific accumulation of these bioactive phenolics in *I. rotunda* under SA and drought stress therefore has dual relevance: not only for medicinal quality but also for the growing demand for natural antioxidant sources in the food industry. This interdisciplinary context underscores the economic and environmental value of preserving phenolic accumulation in woody perennials under climate stress. In recent decades, rising demand for medicinal materials has intensified wild harvesting of *I. rotunda* populations across southern China, while climate change-induced drought episodes increasingly threaten both its natural habitats and cultivated stands [26]. Addressing this challenge will require integrating physiological understanding with modern breeding and management strategies [32].

Exogenous SA has emerged as a promising, environmentally friendly strategy to enhance drought tolerance in various crops [24,33,34], yet its application in medicinal woody species remains largely unexplored. Most investigations of SA-mediated drought tolerance have relied on bulk measurements of total phenolics or antioxidant capacity in leaves, while roots are frequently overlooked despite their central role in water perception and systemic signaling. It remains unclear whether SA enhances drought tolerance primarily by boosting leaf phenolic defenses, by modulating root metabolism, or by coordinating complementary responses across both organs [35,36]. In particular, how exogenous SA affects the qualitative and quantitative profiles of individual phenolic metabolites in leaves versus roots under drought, and which specific compounds are associated with mitigation of oxidative damage, has not been systematically addressed [3]. Addressing these questions is essential for developing evidence-based cultivation protocols that sustain both stress resilience and the accumulation of pharmaceutically valuable metabolites in *I. rotunda*.

In this study, we investigated how exogenous SA modulates drought responses in *I. rotunda* with a focus on tissue-specific phenolic metabolism. We hypothesized that SA alleviates drought-induced oxidative damage through distinct yet coordinated metabolic adjustments in leaves and roots. The specific objectives were: (i) to determine whether exogenous SA mitigates drought-induced oxidative damage at the whole-plant and tissue levels; (ii) to characterize how drought, SA, and their combination reshape phenolic profiles and antioxidant capacity in leaves versus roots; and (iii) to identify key phenolic metabolites associated with stress mitigation and tissue-specific protection. By integrating physiological measurements with high-resolution metabolomics, this work provides the first tissue-resolved insight into SA-mediated phenolic reprogramming in a woody medicinal species, advancing both mechanistic understanding and practical strategies for improving stress resilience and phytochemical quality.

## 2. Materials and Methods

### 2.1. Plant Material and Experimental Design

Ten-month-old seedlings of Kurogane holly (*Ilex rotunda* Thunb.) were obtained from the Winterberry Base of Runtu Horticulture Technology Co., Ltd. (Yuhang District, Hangzhou, China). The experiment was conducted in a completely randomized design (CRD) in a greenhouse at Zhejiang A&F University, Hangzhou, China, during the growing season (September 2025). Plants were transplanted into plastic pots (9 cm diameter × 12 cm height) filled with a homogenized substrate mixture of peat, sand, and soil (1:1:1, *v*/*v*/*v*) and acclimatized for one month in a controlled greenhouse environment maintained at 22 ± 2 °C with a 12 h photoperiod and 65–70% relative humidity. During acclimation, plants were irrigated every three days.

Following acclimatization, plants of uniform size (approximately 45 cm in height) were selected and randomly assigned to four treatment groups (*n* = 6 biological replicates per group): (i) well-watered control (CK), (ii) drought stress (D; water withheld for 10 days), (iii) salicylic acid treatment (SA; normal irrigation with foliar SA application), and (iv) combined drought and salicylic acid treatment (DSA; water withheld with foliar SA application). While seedlings of woody perennials are genetically heterogeneous, the random assignment of uniformly sized plants to treatment groups minimizes systematic bias. The consistency of responses across biological replicates supports the robustness of the observed treatment-specific effects, which was the primary focus of this study. To balance analytical requirements with statistical robustness, three replicates per treatment were randomly selected for biochemical assays and metabolomic profiling, with each sample analyzed in technical triplicates; the remaining three replicates were used for phenotypic documentation and gravimetric soil water content determination.

### 2.2. Salicylic Acid Application, Drought Treatment, and Sample Harvesting

A 500 mM stock solution of salicylic acid (CAS No. 69-72-7) was prepared in ethanol. A preliminary dose–response experiment (0, 50, 100, and 200 µM SA) was conducted. Based on visual assessment, 200 µM SA caused mild leaf chlorosis, while 50 µM SA provided negligible protection; 100 µM SA was therefore selected for all subsequent treatments, an effective dose previously reported in woody species [3,37]. The 100 µM SA working solution was freshly prepared before each application by diluting the stock solution with distilled water containing 0.1% (*v*/*v*) Tween-20 (CAS No. 9005-64-5; Shanghai Yuanye Bio-Technology Co., Ltd., Shanghai, China).

Plants in the SA and DSA groups were sprayed to run-off on days 0, 4, and 8 of the 10-day experimental periods. This application schedule was designed to maintain physiologically active SA levels throughout the drought duration, based on previously reported foliar uptake kinetics in woody species [3,38]. Control and drought-only plants were sprayed with an equivalent solution containing the same concentrations of ethanol and Tween-20 but without SA. Control plants (CK and SA) were irrigated every four days throughout the experimental period, while drought stress was imposed by completely withholding water from the D and DSA groups for 10 days. Soil water content (SWC) was monitored daily using the gravimetric method. Fresh soil samples (approximately 10 g) were collected from each pot, weighed to obtain fresh weight, dried at 105 °C to constant mass in a forced-air drying oven (DHG-9240A, Shanghai Jinghong Experimental Equipment Co., Ltd., Shanghai, China), and reweighed to determine dry weight. SWC (%) was calculated as [(fresh weight − dry weight)/fresh weight] × 100.

After 10 days, when drought-induced symptoms (e.g., leaf wilting) became clearly evident, leaf and root tissues were harvested from three randomly selected biological replicates per treatment. All samples were immediately flash-frozen in liquid nitrogen and stored at −80 °C for subsequent biochemical and metabolomic analyses. At harvest, soil water content was 67.6% for CK, 23.9% for D, and 24.4% for DSA, confirming the establishment of severe and uniform drought stress across treated groups. Field capacity was determined separately (*n* = 5 pots) using the standard method (saturation for 24 h followed by 48 h drainage) [3]. The drought treatment (SWC = 23.9 ± 1.67%) corresponded to approximately 73.5% of field capacity (32.5%).

### 2.3. Determination of Photosynthetic Pigments

For pigment analysis, fresh leaf tissue (approximately 0.1 g) was ground in liquid nitrogen and extracted with 95% ethanol in darkness at 25 °C for 24 h. The extract was centrifuged at 5000× *g* for 10 min, and the supernatant was collected. Absorbance was measured at 665, 649, and 470 nm using a SPARK multimode microplate reader (TECAN, Zurich, Switzerland) against a 95% ethanol blank. Chlorophyll a and chlorophyll b contents were calculated using standard equations [3,39,40] and expressed as mg/g fresh weight.

### 2.4. Quantification of Total Phenolics and Radical Scavenging Assay in Leaf and Root Tissues

Total phenolic content and DPPH radical scavenging capacity were determined in both leaf and root tissues using commercial assay kits (Suzhou Michy Biomedical Technology Co., Ltd., Suzhou, China). Fresh tissue samples (0.1 g) were homogenized according to the manufacturer’s protocols for each kit. Total phenolic content was measured at 700 nm using the Total Phenolic Compounds Assay Kit (Catalog No. Mo119A), following the principle of the Folin–Ciocalteu method [41], and expressed as mg gallic acid equivalents per gram fresh weight (mg GAE g^−1^ FW). 2,2-diphenyl-1-picrylhydrazyl (DPPH) free radical scavenging capacity was evaluated at 515 nm employing the Total Antioxidant Capacity (T-AOC) Assay Kit (Catalog No. M0112A), based on the original DPPH radical scavenging protocol [42]. Hydroxyl radical (•OH) scavenging rate (%) was determined at 510 nm using the Hydroxyl Radical Scavenging Assay Kit (Catalog No. M0116A) following the manufacturer’s instructions. All absorbance measurements were performed on a SPARK multimode microplate reader (TECAN, Zurich, Switzerland).

### 2.5. Assessment of Oxidative Stress Markers in Leaf and Root Tissues

Hydrogen peroxide (H_2_O_2_), superoxide anion (O_2_·^−^), and malondialdehyde (MDA) contents were quantified in both leaf and root tissues using Suzhou Michy Biomedical Technology Co., Ltd., commercial assay kits (Suzhou, China). Fresh tissue samples (0.1 g) were homogenized and processed following the respective kit protocols. H_2_O_2_ concentration was measured at 415 nm with the Hydrogen Peroxide Assay Kit (Catalog No. M0107A), superoxide anion production rate was determined at 530 nm by using Superoxide Anion Assay Kit (Catalog No: M0114A), and lipid peroxidation was assessed at 532 nm using the Lipid Peroxidation Assay Kit (Catalog No. M0106A). Absorbance readings were obtained using a SPARK multimode microplate reader (TECAN, Switzerland).

### 2.6. UPLC–MS/MS-Based Profiling of Phenolic Compounds in Leaf and Root Tissues

Phenolic metabolite profiling of *Ilex rotunda* leaf and root tissues was conducted using an ultra-performance liquid chromatography–tandem mass spectrometry (UPLC–MS/MS) platform at Wuhan Metware Biotechnology Co., Ltd. (Wuhan, China). The system comprised an ExionLC™ AD UPLC (SCIEX, Marlborough, MA, USA) coupled to a QTRAP^®^ 6500+ triple quadrupole-linear ion trap mass spectrometer (SCIEX). Freeze-dried tissue samples were finely ground, and 30 mg of powder was extracted with 600 µL of pre-chilled methanol:water (7:3, *v*/*v*) containing 2-chlorophenylalanine (1 µg/mL) as an internal standard. The mixture was vortexed, ultrasonicated in an ice-water bath for 30 min, and centrifuged at 13,000 rpm for 20 min at 4 °C. The supernatant was filtered through a 0.22 µm membrane prior to analysis.

Chromatographic separation was performed on an Agilent SB-C18 column (2.1 × 100 mm, 1.8 µm) maintained at 40 °C. The mobile phase consisted of 0.1% formic acid in water (solvent A) and acetonitrile (solvent B). A linear gradient elution was applied from 5% to 95% solvent B over 9 min at a flow rate of 0.35 mL min^−1^. Data acquisition was performed in scheduled multiple reaction monitoring (MRM) mode. Ion source parameters were optimized as follows: ion spray voltages +5500 V (ESI^+^) and −4500 V (ESI^−^), source temperature 500 °C, curtain gas 25 psi, and nebulizer gases GS1 and GS2 set to 50 psi and 60 psi, respectively.

For each metabolite, precursor-to-product ion transitions, declustering potentials, and collision energies were optimized using authentic standards and the Metware Database (MWDB). Compound identification was based on alignment of retention times, fragmentation patterns, and MS/MS spectra with reference database entries [43]; confidence levels are provided in Supplementary Table S1. Three independent biological replicates per treatment (CK, D, SA, and DSA) were analyzed for both leaf and root tissues.

### 2.7. Differential Metabolite Identification and Visualization

Differentially accumulated metabolites (DAMs) were identified using dual criteria: |log_2_FC| ≥ 1.0 and VIP > 1.0, with statistical significance determined by Student’s *t*-test (*p* < 0.05) followed by FDR correction [44]. Venn diagrams of DAMs were generated using the EVenn online platform (https://www.bic.ac.cn/EVenn/#/, accessed on 11 April 2026) [45].

For visualization, bar plots of log_2_FC values for the top 20 DAMs were generated for each pairwise comparison using (R v3.5.1) ggplot2 (v3.3.0). For cross-tissue comparisons (CKL vs. CKR, DL vs. DR, SAL vs. SAR, DSAL vs. DSAR), lollipop plots of the top 50 metabolites (|log_2_FC|, VIP > 1.0, *p* < 0.05) were constructed, where bar length represents |log_2_FC|, circle size represents VIP, and color indicates direction (red: higher in leaves; blue: higher in roots). Significance levels: *** *p* < 0.001, ** *p* < 0.01, * *p* < 0.05 [44]. All visualizations were generated using R with ggplot2 and ComplexHeatmap packages [46].

### 2.8. OPLS-DA Modeling

Orthogonal partial least squares discriminant analysis (OPLS-DA) was conducted using the MetaboAnalystR package (v1.0.1) in R (v3.5.1), to identify metabolites discriminating treatment groups and tissues [47]. Model validity was assessed through 200 permutation tests; models were considered valid when original R^2^Y and Q^2^ values exceeded all permuted values (*p* < 0.05). Variable importance in projection (VIP) scores were calculated, with VIP > 1.0 considered significant. S-plots combining covariance (p [1]) and correlation (p(corr)[1]) were generated; metabolites with |p[1]| > 0.05 and |p(corr)[1]| > 0.5 were considered significant contributors.

### 2.9. Statistical Analysis

All statistical analyses were performed using R software (version 4.2.0). Prior to Duncan’s multiple range test, assumptions of normality and homogeneity of variance were verified; data transformations were applied where necessary. Duncan’s multiple range test was used to assess significant differences among treatments (*p* < 0.05). For metabolomic data, FDR correction was applied using the p.adjust function in R (stats package) with the Benjamini–Hochberg method [44]. Data are presented as mean ± SD from three biological replicates.

For metabolomic data, raw peak areas were normalized using unit variance scaling. Principal component analysis (PCA) and hierarchical cluster analysis (HCA; Euclidean distance, Ward’s linkage) were performed using R stats package (v3.5.1) [46]. Circular visualizations were generated using circlize (v0.4.15). Pearson correlation coefficients were calculated and visualized as combined heatmap and scatter plot matrices using the ComplexHeatmap package.

## 3. Results

### 3.1. Physiological and Metabolic Responses

**Figure 1:**
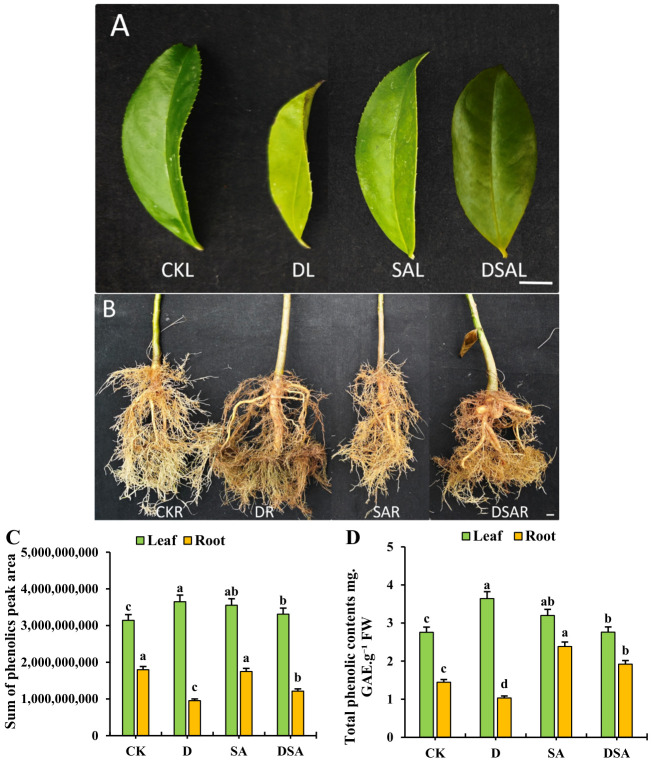
Phenotypic responses and total phenolic content of *Ilex rotunda* under drought and salicylic acid treatments. (**A**) Leaf and (**B**) root phenotypes of control (CK), drought (D), salicylic acid (SA), and combined drought + salicylic acid (DSA) treated plants (line denotes 1 cm). (**C**) Total phenolic content expressed as the sum of raw peak areas (arbitrary units) of all identified compounds. (**D**) Total phenolic content quantified spectrophotometrically and expressed as mg GAE g^−1^ fresh weight (FW). Bars represent mean ± standard deviation of three biological replicates (*n* = 3). Different letters indicate significant differences among treatments within each tissue (Duncan’s Multiple Range Test, *p* < 0.05, FDR-corrected).

Under control conditions, *Ilex rotunda* leaves appeared healthy and green, while roots exhibited normal light brown coloration (Figure 1A,B). Drought stress induced visible leaf yellowing and dark brown root discoloration. SA treatment alone maintained relatively greener leaves and lighter brown roots, while combined DSA treatment partially alleviated drought symptoms, suggesting a protective effect of SA against drought-induced damage.

Total phenolic content, estimated as sum of raw peak areas of all identified compounds, revealed tissue-specific responses to treatments (Figure 1C). In leaves, drought increased phenolic accumulation (3.64 mg GAE g^−1^ FW) compared to control (2.76), whereas DSA treatment reduced leaf phenolics to control levels (2.76), indicating that SA reversed the drought-induced phenolic increase (Figure 1D). In roots, drought decreased phenolic concentration (1.03) relative to control (1.45), while DSA treatment partially restored root phenolics (1.92) (Figure 1D). Thus, under drought, SA treatment reprogrammed phenolic distribution—reducing leaf phenolic accumulation while partially restoring root phenolic pools. The minor discrepancy between semi-quantitative peak areas and absolute quantification (mg g^−1^ FW) reflects inherent methodological differences: peak area depends on compound-specific MS response, whereas mg g^−1^ FW provides a spectrophotometrically standardized measure of total phenolics. Both metrics independently confirm the observed tissue-specific phenolic shifts.

**Figure 2:**
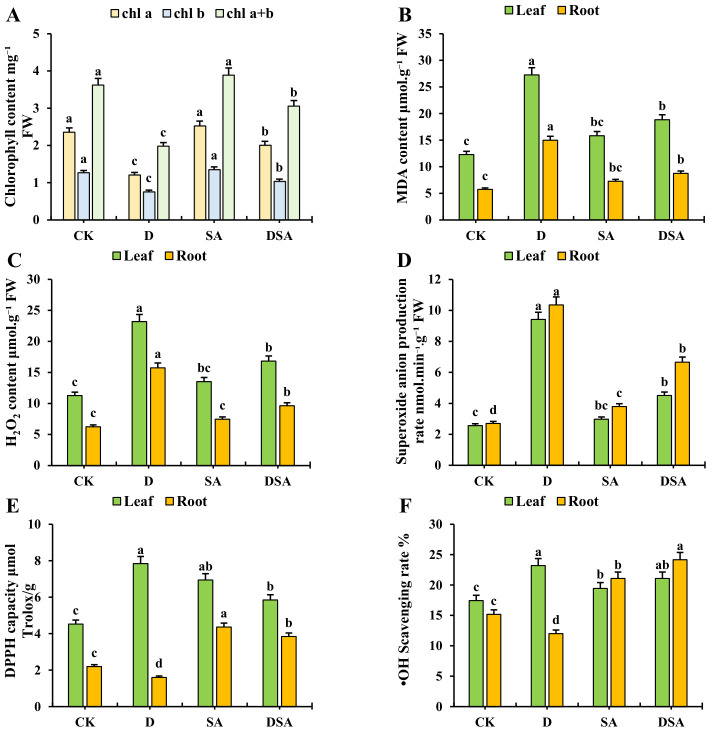
Physiological and oxidative stress parameters in *Ilex rotunda* leaves and roots under control (CK), drought (D), salicylic acid (SA), and combined drought + salicylic acid (DSA) treatments. (**A**) Chlorophyll a, b, and a + b content (mg g^−1^ FW) measured in leaf tissues only. (**B**) Malondialdehyde (MDA) (μmol g^−1^ FW). (**C**) Hydrogen peroxide (H_2_O_2_) (μmol g^−1^ FW). (**D**) Superoxide anion production rate (nmol min^−1^ g^−1^ FW). (**E**) DPPH radical scavenging capacity (μmol Trolox g^−1^). (**F**) Hydroxyl radical scavenging rate (%). Bars represent mean ± SD (*n* = 3). Different small letters indicate significant differences among treatments within each tissue (Duncan’s Multiple Range Test, *p* < 0.05).

Drought stress significantly reduced chlorophyll a, b, and total chlorophyll in leaves (48.4%, 39.7%, and 45.3% decrease, respectively), while SA alone maintained levels similar to control and DSA partially restored chlorophyll content (Figure 2A). Drought induced oxidative damage in both tissues, with MDA, H_2_O_2_, and superoxide anion increasing 2.2-, 2.1-, and 3.7-fold in leaves, and 2.6-, 2.5-, and 3.8-fold in roots (FDR-adjusted *p* < 0.05), respectively (Figure 2B–D). Superoxide production was consistently higher in roots than leaves, indicating tissue-specific stress responses. SA treatment alone caused mild increases in oxidative markers (priming-like effect), while DSA significantly reduced all oxidative parameters compared to drought alone, confirming SA mitigates drought-induced oxidative damage. These results corroborate the phenotypic damage observed in Figure 1A,B.

Antioxidant capacity showed tissue-specific patterns (Figure 2E,F). In leaves, drought increased DPPH and hydroxyl radical scavenging rate by 73.4% and 33.0%, respectively, consistent with elevated phenolic content (Figure 1C,D). In roots, drought decreased DPPH scavenging (27.1% reduction) but increased hydroxyl radical scavenging rate (26.3% increase), suggesting differential antioxidant deployment. SA treatment enhanced both antioxidant parameters in roots under drought, with DSA showing the highest hydroxyl radical scavenging rate 59.3% higher than drought alone. The enhanced antioxidant capacity under SA and DSA treatments aligns with the mitigated stress phenotypes and the phenolic reprogramming patterns observed in Figure 1.

### 3.2. Hierarchical Cluster Analysis of Phenolic Compounds

**Figure 3:**
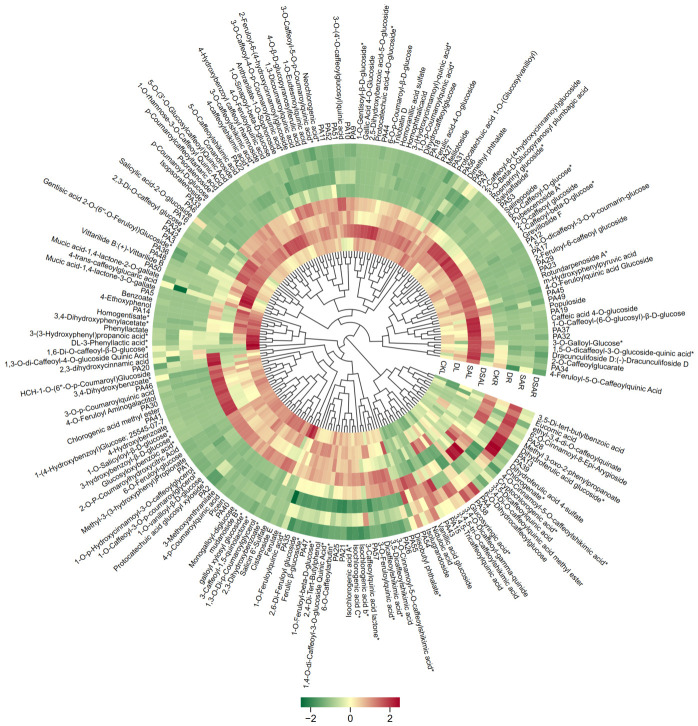
Hierarchical cluster analysis of phenolic compounds in *Ilex rotunda* leaves and roots. Heatmap shows normalized abundance of compounds (rows) across eight sample groups (columns). * Means isomers. Color scale: low (green) to high (red). Sample groups: control (CKL), drought (DL), salicylic acid (SAL), combined drought + salicylic acid leaves (DSAL); control (CKR), drought (DR), salicylic acid (SAR), combined drought + salicylic acid roots (DSAR).

Hierarchical cluster analysis (HCA) of phenolic compounds revealed tissue type as the primary driver of metabolic profiles, with leaf (CKL, DL, SAL, DSAL) and root (CKR, DR, SAR, DSAR) samples forming distinct major branches (Figure 3). Within leaves, control samples (CKL) separated from drought (DL) treatment while combined treatment leaves (DSAL) formed a distinct subcluster, indicating unique metabolic reprogramming under dual stress. In roots, control (CKR) and SA-treated (SAR) samples grouped together, whereas drought (DR) and combined treatment roots (DSAR) formed a separate subcluster, demonstrating that drought imposes a stronger metabolic signature than SA alone.

The heatmap revealed tissue-enriched metabolite modules. Caffeoylquinic acid derivatives (e.g., 3,4-Dicaffeoylquinic acid, 5-O-Caffeoylshikimic acid) accumulated preferentially in leaves, particularly under DSAL treatment. Conversely, 3,5-Dicaffeoylquinic acid and sulfated benzoates enriched in roots, with strongest signals in DSAR samples. Notably, 4-(2,6-Dihydroxybenzoyl)-3-Formyl-5-Hydroxybenzoic acid accumulated specifically in SAL and DSAL but was nearly absent in roots, while 3,5-Dicaffeoylquinic acid was strongly induced in DSAR with minimal leaf expression. These patterns validate that phenolic metabolism in *Ilex rotunda* is differentially regulated across tissues and in response to drought, SA, and their combination.

### 3.3. Tissue- and Treatment-Specific Metabolite Profiles

**Figure 4:**
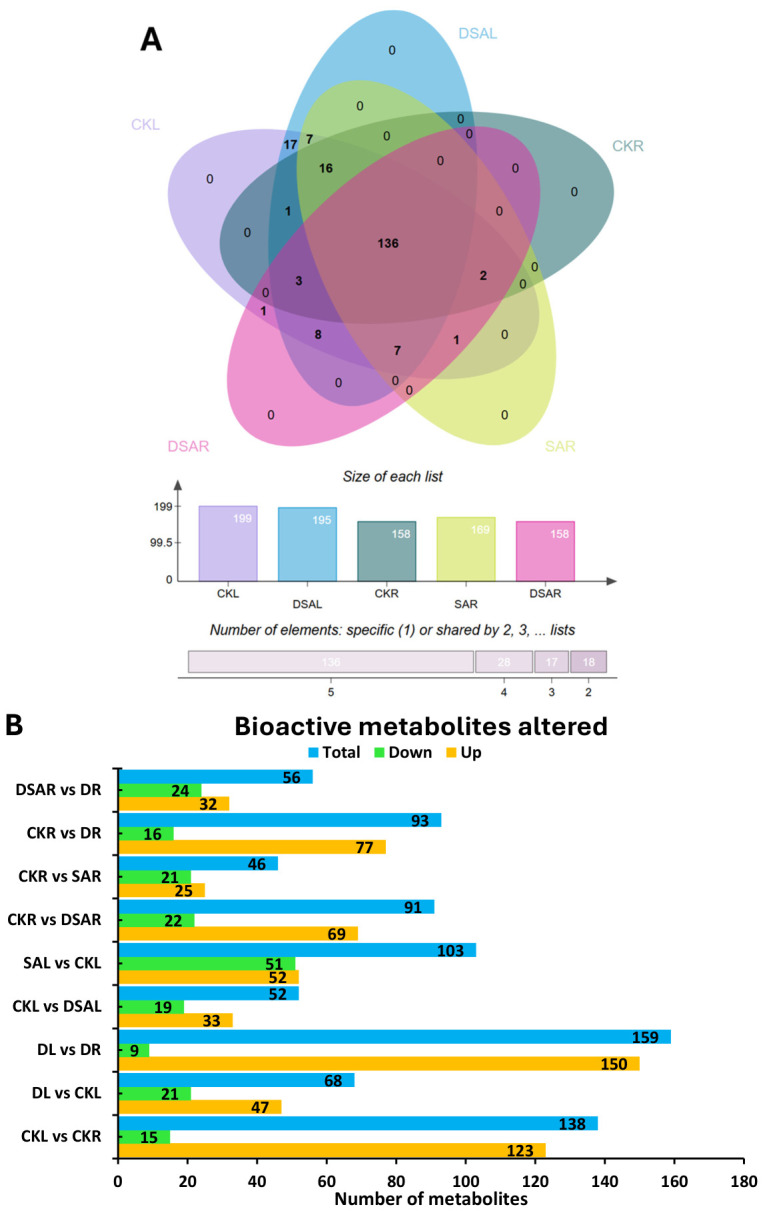

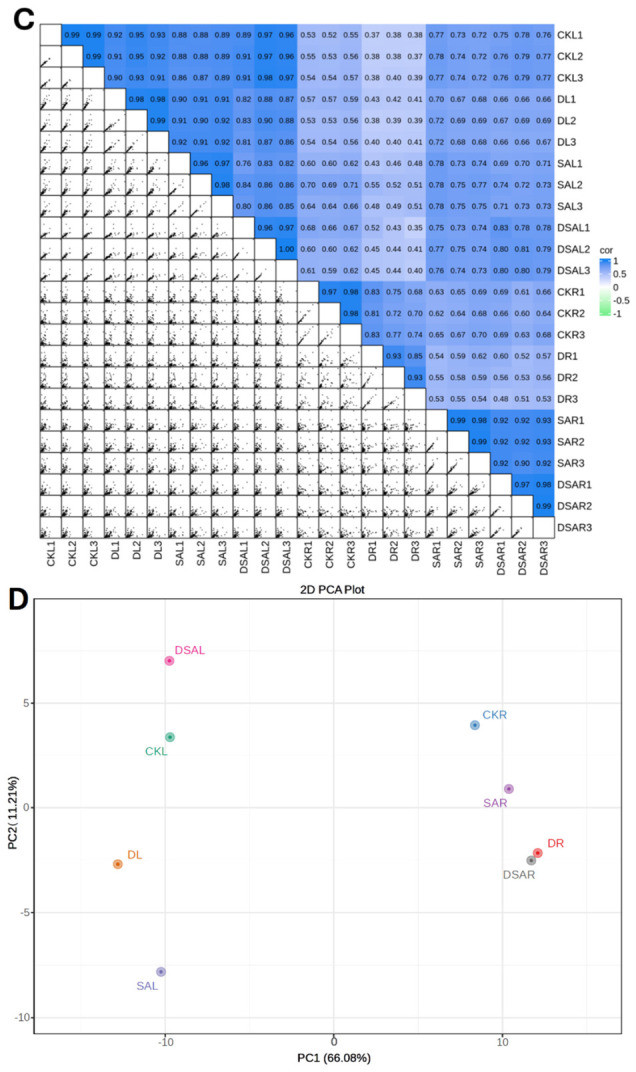
Multivariate analysis of phenolic compounds in *Ilex rotunda* leaves and roots. (**A**) Venn diagram showing metabolite distribution among six treatment comparisons. (**B**) Differentially accumulated metabolites for key comparisons (VIP > 1, |log_2_FC| > 2). (**C**) Pearson correlation heatmap of all samples. (**D**) PCA score plot. Abbreviations: CKL (control leaf), DL (drought leaf), SAL (salicylic acid leaf), DSAL (drought + salicylic acid leaf), CKR (control root), DR (drought root), SAR (salicylic acid root), DSAR (drought + salicylic acid root). In (**D**), the PCA score plot reveals a clear separation of leaf and root samples along PC1, highlighting tissue identity as the dominant source of phenolic variation; separation along PC2 further discriminates among treatments, reflecting the distinct metabolic reprogramming induced by drought, SA, and their combination.

Venn diagram showing the distribution of differentially accumulated metabolites among six treatment comparisons (Figure 4A). A core set of 136 compounds was commonly detected across all six groups. Individual groups showed distinct total metabolite counts: CKL (199 compounds), DSAL (195), CKR (158), SAR (169), and DSAR (158). Overlap analysis revealed that 18 compounds were shared between two groups, 17 compounds among three groups, and 28 compounds among four groups, indicating substantial metabolic overlap across tissues and treatments. These variable compounds among groups reflect treatment- and tissue-specific metabolic adjustments.

Comparative analysis of differentially accumulated phenolic compounds (VIP > 1, |log_2_FC| > 2) revealed distinct tissue- and treatment-specific responses (Figure 4B). Between control leaves and roots (CKL vs. CKR), 138 metabolites were differentially accumulated, with 123 compounds upregulated in leaves, establishing tissue-specific metabolic baselines. In leaves, drought (DL vs. CKL) induced 68 differentially accumulated metabolites (47 up, 21 down), while salicylic acid (SAL vs. CKL) resulted in 103 (52 up, 51 down). Combined treatment (DSAL vs. CKL) produced only 52 (33 up, 19 down). In roots, drought (DR vs. CKR) elicited 93 differentially accumulated metabolites (77 up, 16 down), SA (SAR vs. CKR) induced 46 (25 up, 21 down), and combined treatment (DSAR vs. CKR) showed 91 (69 up, 22 down), indicating drought dominates root metabolic responses.

Cross-tissue comparisons revealed 159 differentially accumulated metabolites between drought leaves and roots (DL vs. DR; 150 up in leaves) (Figure 4B). Pearson correlation showed high replicate reproducibility (r > 0.97) and clear tissue separation: leaf samples intercorrelated strongly (r = 0.80–0.99), as did roots (r > 0.92), while leaf-root correlations were low (r = 0.37–0.83) (Figure 4C). PCA confirmed these patterns, with PC1 (66.08%) separating tissues and PC2 (11.21%) resolving treatment effects (Figure 4D). Within leaves, CKL and DL clustered together, while SAL and DSAL shifted along PC2. Within roots, CKR and SAR grouped together, while DR and DSAR separated along PC2. These results showed treatment- and tissue-specific metabolic reprogramming.

### 3.4. OPLS-DA Modeling Identifies Tissue-Specific Biomarkers

**Figure 5:**
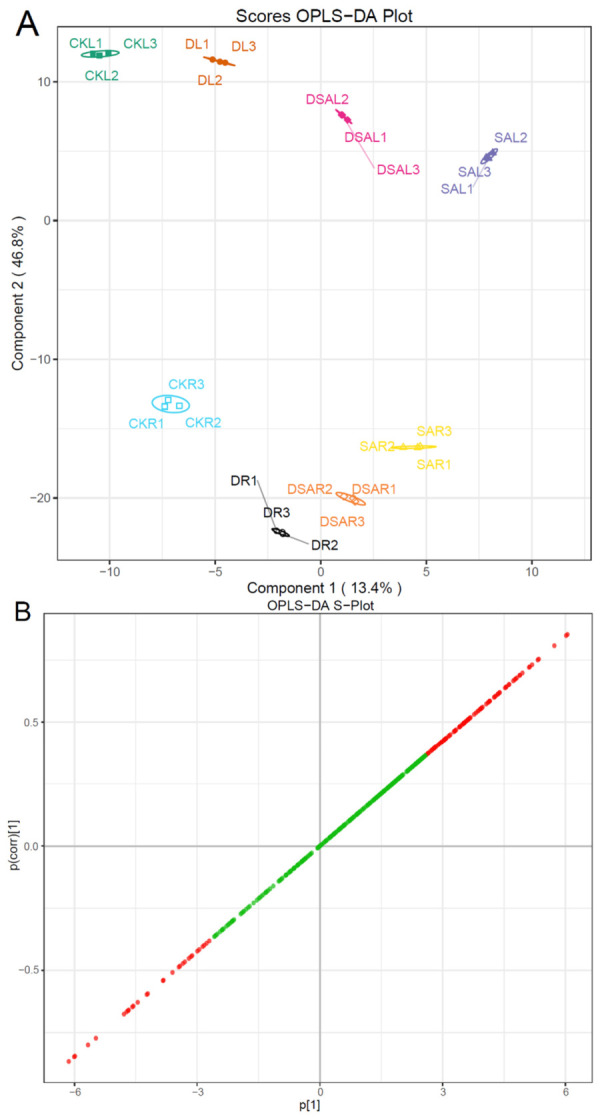

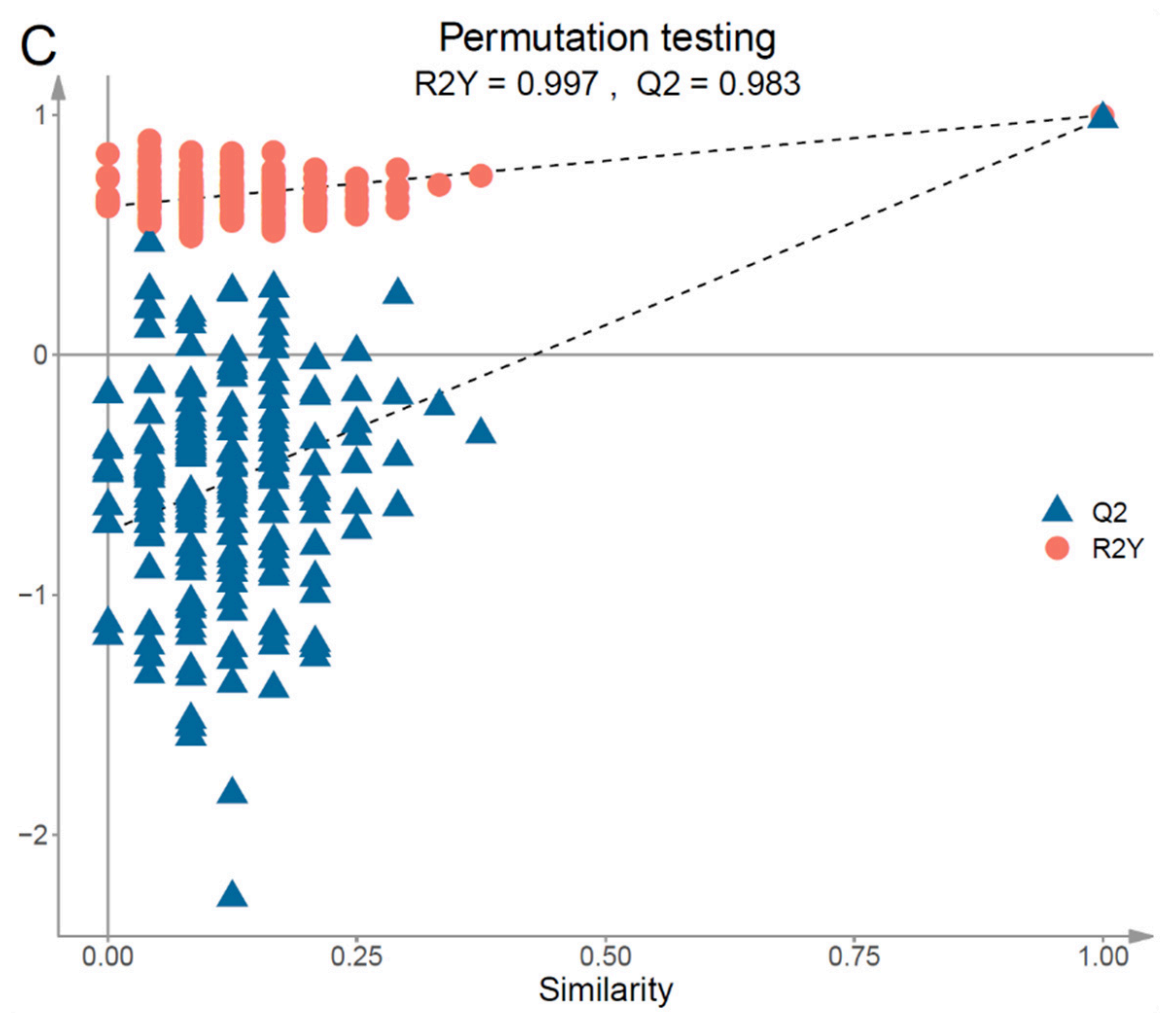
OPLS-DA analysis of phenolic compounds in *Ilex rotunda* leaves and roots under control and stress treatments. (**A**) OPLS-DA score plot of all treatment groups. Abbreviation: control leaves (CKL), drought leaves (DL), salicylic acid leaves (SAL), combined drought + salicylic acid leaves (DSAL), control roots (CKR), drought roots (DR), salicylic acid roots (SAR), and combined drought + salicylic acid roots (DSAR). (**B**) OPLS-DA S-plot showing metabolites contributing to group separation. (**C**) Permutation test (200 permutations) validating model robustness. Orange and blue points represent R^2^Y and Q^2^ values from permuted models, with corresponding regression lines (dashed).

OPLS-DA of all eight treatment groups showed clear tissue separation along the first predictive component, confirming PCA results (Figure 5A). In leaves, SAL and DSAL clustered separately from CKL and DL, indicating SA-driven metabolic reprogramming. In roots, DR and DSAR diverged from CKR and SAR, demonstrating that drought dominates root metabolic responses. The S-plot identified metabolites with high discrimination potential (|p[1]| > 0.05, |p(corr)[1]| > 0.5) as candidate indicators for tissue- and treatment-specific responses (Figure 5B). Red points denote metabolites with VIP > 1. Permutation testing (200 iterations) confirmed model robustness (R^2^Y = 0.997, Q^2^ = 0.983; *p* < 0.005) (Figure 5C). These results validate the robustness of the OPLS-DA model and identify key phenolic biomarkers distinguishing tissue- and treatment-specific metabolic responses in *Ilex rotunda*.

### 3.5. Treatment-Specific Phenolic Reprogramming Reveals Distinct Stress Response Signatures in Leaves and Roots

**Figure 6:**
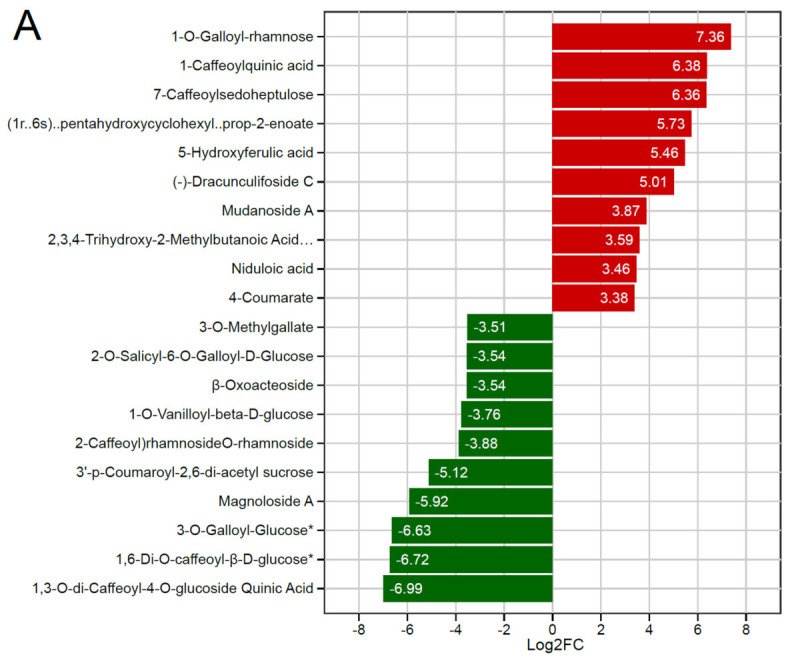

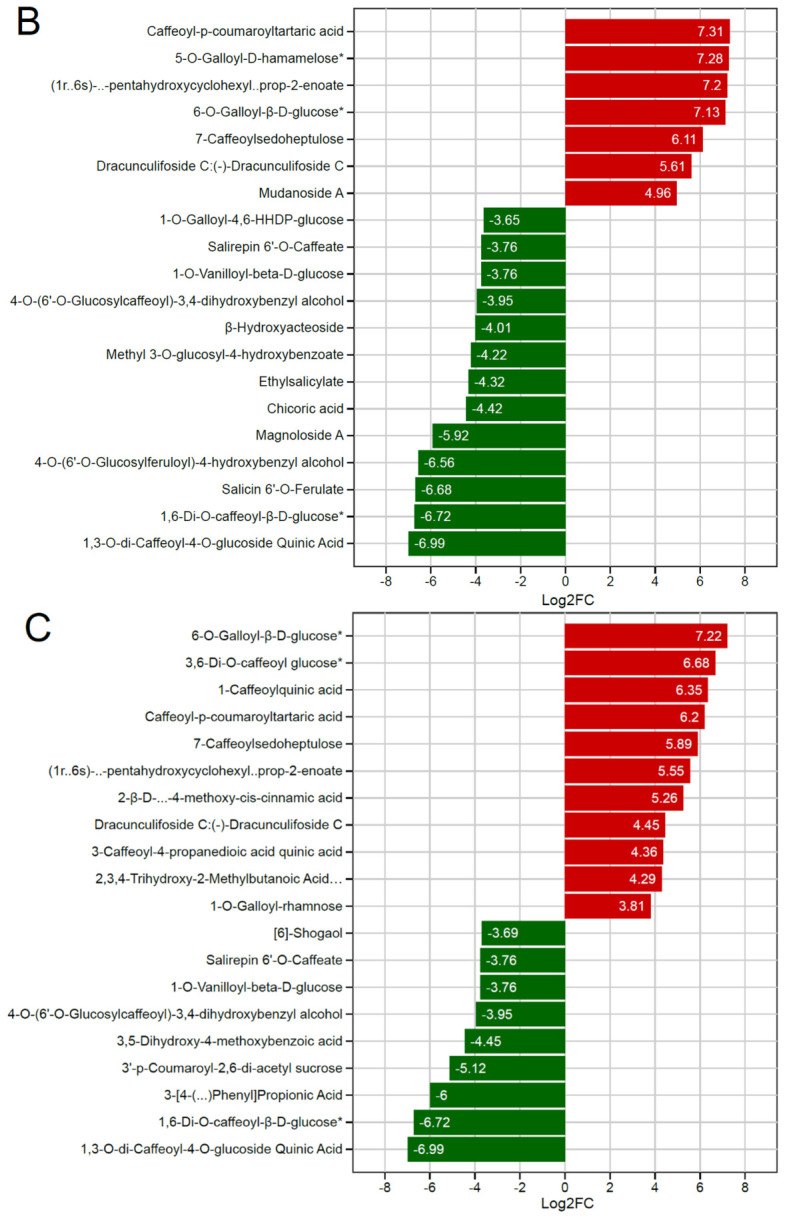

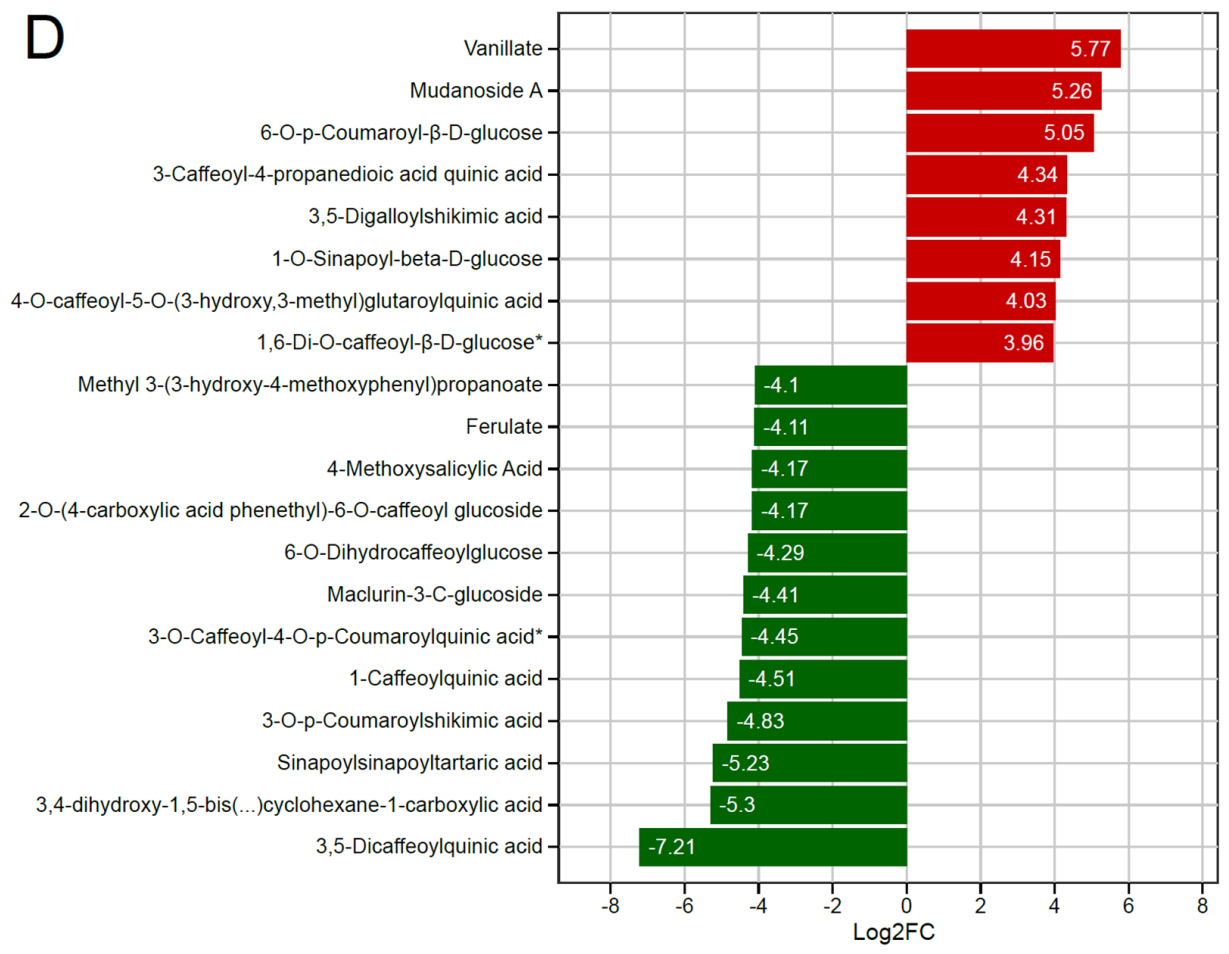
Top 20 differentially accumulated phenolic compounds in *Ilex rotunda* leaves and roots under stress treatments. Metabolite with dots ‘…’ their full names are provided in supplementary tables. * Means isomers. Bar plots show log_2_FC values for significantly altered compounds (VIP > 1.0, *p* < 0.05) in: (**A**) DL vs. CKL, (**B**) SAL vs. CKL, (**C**) DSAL vs. CKL, and (**D**) DR vs. CKR. Positive/negative values indicate up-/down-regulation under treatment.

To pinpoint metabolites responsible for treatment- and tissue-specific clustering, the top 20 differentially accumulated compounds (|log_2_FC|) were examined (Figure 6). In leaves, drought stress (DL vs. CKL) induced pronounced reprogramming of 1-O-Galloyl-rhamnose (log_2_FC = 7.36), 1-Caffeoylquinic acid (6.38), and 7-Caffeoylsedoheptulose (6.36), while strongly suppressing 1,3-O-di-Caffeoyl-4-O-glucoside quinic acid (−6.99), 1,6-Di-O-caffeoyl-β-D-glucose (−6.72), and 3-O-Galloyl-glucose (−6.63) (Figure 6A; Supplementary Table S2). This bidirectional regulation suggests active metabolic reconfiguration under drought, with selective reprogramming of specific caffeoyl conjugates and galloyl derivatives.

Salicylic acid treatment (SAL vs. CKL) elicited a distinct bidirectional reprogramming pattern (Figure 6B; Supplementary Table S3). The most highly up-accumulated compounds included Caffeoyl-p-coumaroyltartaric acid (7.31), 5-O-Galloyl-D-hamamelose (7.28), and (1r,…,6s)-pentahydroxycyclohexyl prop-2-enoate (7.20). However, in contrast to the initial observation, SA treatment also induced substantial down-reprogramming of several phenolic compounds, most notably 1,3-O-di-Caffeoyl-4-O-glucoside quinic acid (−6.99), 1,6-Di-O-caffeoyl-β-D-glucose (−6.72), and Salicin 6′-O-Ferulate (−6.68). This bidirectional response indicates that SA simultaneously activates and suppresses distinct branches of phenolic metabolism, with selective reprogramming of specific galloyl and caffeoyl conjugates alongside repression of particular caffeoyl-glucose and quinic acid derivatives.

The combined treatment (DSAL vs. CKL) produced an intermediate signature blending drought and SA features (Figure 6C; Supplementary Table S4). Highly up-accumulated compounds included 6-O-Galloyl-β-D-glucose (7.22), 3,6-Di-O-caffeoyl glucose (6.68), and 1-Caffeoylquinic acid (6.35), while the most suppressed metabolites includes 1,3-O-di-Caffeoyl-4-O-glucoside quinic acid (−6.99) and 1,6-Di-O-caffeoyl-β-D-glucose (−6.72), which mirrored those down-accumulated under drought alone. This suggests that drought-driven suppression of specific caffeoyl conjugates persists even in the presence of SA.

In roots, drought stress (DR vs. CKR) induced a strikingly different response profile (Figure 6D; Supplementary Table S5). The most highly up-accumulated compounds were Vanillate (5.77), Mudanoside A (5.26), and 6-O-p-Coumaroyl-β-D-glucose (5.05), while the strongest suppression targeted 3,5-Dicaffeoylquinic acid (−7.21), sinapoylsinapoyltartaric acid (−5.23), and 3-O-p-Coumaroylshikimic acid (−4.83). The pronounced downregulation of multiple caffeoylquinic acid derivatives in roots contrast with their upregulation in drought-treated leaves (e.g., 1-Caffeoylquinic acid), revealing tissue-specific regulation of this compound class. These results reveal tissue- and treatment-specific phenolic reprogramming, with distinct drought and SA signatures and composite responses under combined stress, identifying candidate indicators for metabolic adjustments in *Ilex rotunda*.

### 3.6. Tissue-Specific Phenolic Partitioning Is Constitutively Established and Differentially Amplified by Drought and SA Stress

**Figure 7:**
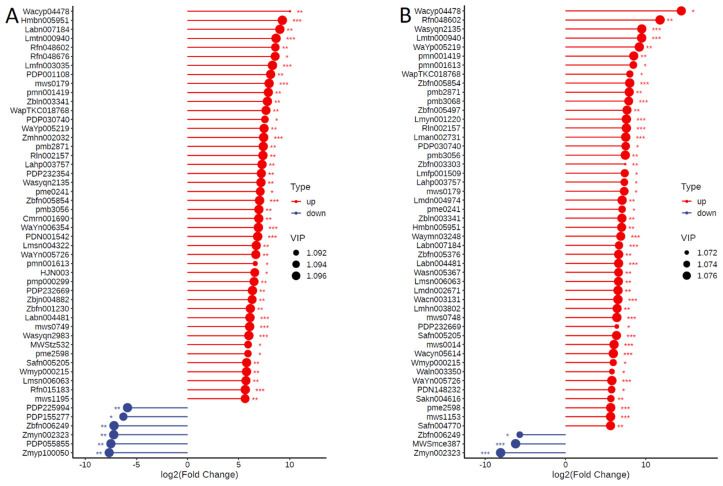

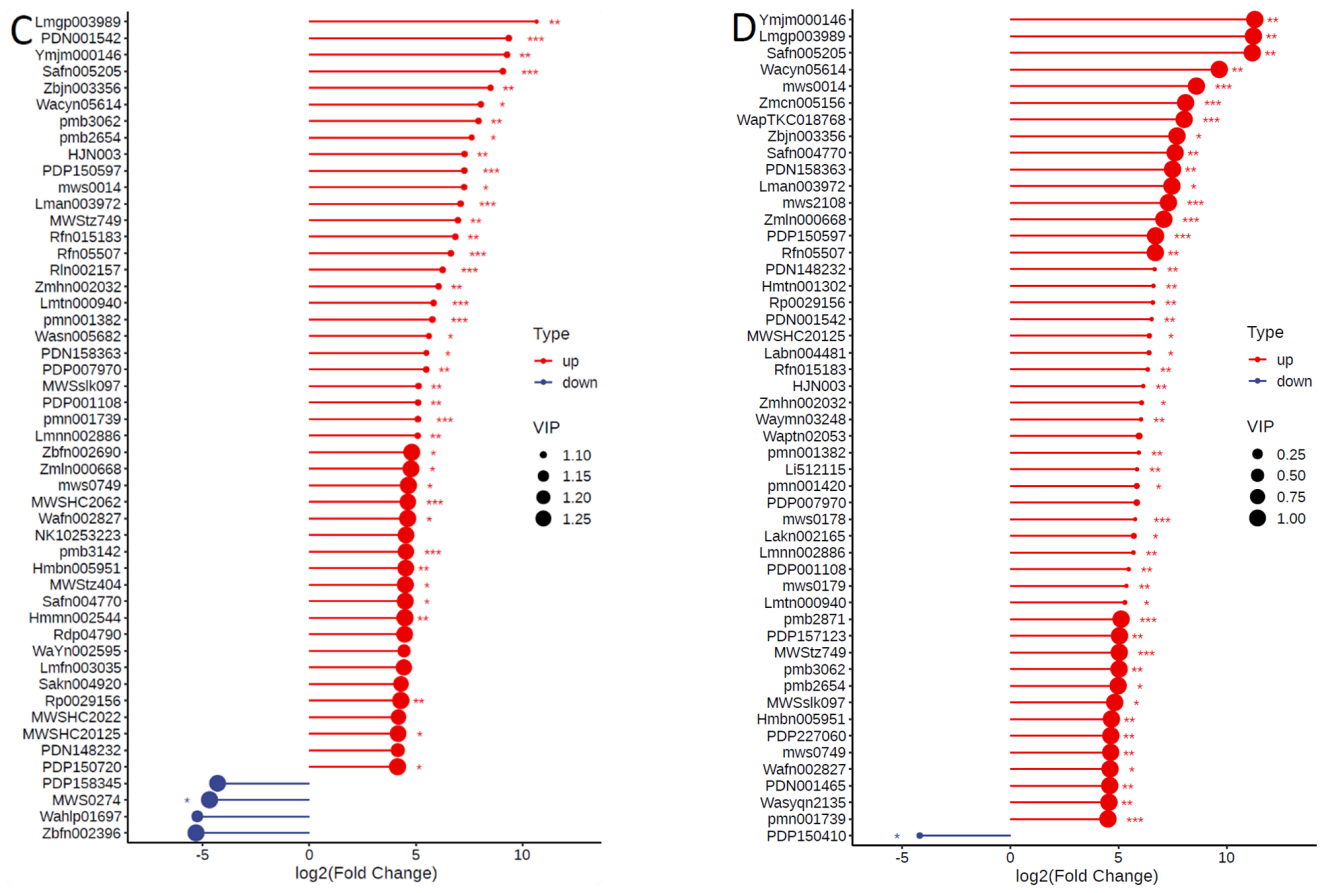
Lollipop plots showing cross-tissue comparison of top 50 significantly accumulated phenolics (|log_2_FC| ≥ 4.0) in *Ilex rotunda* under control and drought stress. (**A**) CKL vs. CKR, (**B**) DL vs. DR, (**C**) SAL vs. SAR, and (**D**) DSAL vs. DSAR. Bar length = |log_2_FC|; dot size = VIP. Positive (red color) log_2_FC indicates higher in leaves and negative (blue color) indicates higher in roots. *** *p* < 0.001, ** *p* < 0.01, * *p* < 0.05.

Comparative analysis of control leaves versus control roots (CKL vs. CKR) revealed profound constitutive differences in phenolic distribution, establishing tissue-specific metabolic baselines (Figure 7A; Supplementary Table S6). Among the top 50 differentially accumulated metabolites, 44 compounds were leaf-enriched, with six compounds preferentially accumulated in roots, including 5-Hydroxyferulic acid (Zmyp100050; log_2_FC = −7.67), Vitexfolin A (PDP055855; −7.50), and 2-Hydroxyphenylacetate (Zmyn002323; −7.22). Leaf tissues exhibited massive enrichment of diverse phenolic conjugates, particularly caffeoyl and galloyl polymers including 1,6-Di-O-caffeoyl-β-D-glucose (Lmsn004322; 6.72) and 1-O-Caffeoyl-4-(4′-Caffeoyl)Caffeoyl Quinic Acid (Zbjn004882; 6.32), establishing leaves as primary hubs for phenolic diversification under constitutive conditions.

Under drought stress (DL vs. DR), this tissue-specific partitioning was profoundly amplified (Figure 7B; Supplementary Table S7). The same root-preferential compounds persisted [2-Hydroxyphenylacetate (Zmyn002323; −8.10) and 3-O-Methylgallate (MWSmce387; −6.24)], indicating conserved root-specific metabolic functions. Drought leaves exhibited extreme enrichment of phenolic conjugates, with log_2_FC values exceeding 14 [4-(2,6-Dihydroxybenzoyl)-3-Formyl-5-Hydroxybenzoic acid (Wacyp04478; 14.41)], compared to 10.02 under control conditions. Multiple compound classes, including caffeoyl conjugates [p-Coumaroyl-D-glucose (pmn001419; 8.49)], galloylated derivatives [Gallic Acid 4-O-Glucoside (Wasyqn2135; 9.49)], and complex phenolic polymers, became dramatically more leaf-preferential under drought.

SA treatment (SAL vs. SAR) similarly induced pronounced leaf-preferential reprogramming, with distinct compound classes responding (Figure 7C; Supplementary Table S8). The most highly leaf-enriched compounds included dicaffeoylshikimic acid (Lmgp003989; 10.66), 2-O-caffeoyl glucoside (PDN001542; 9.36), and p-Hydroxyphenyl 6-O-(E)-caffeoyl-β-D-glucopyranoside (Ymjm000146; 9.27). Notably, caffeoylquinic acid derivatives including 3-O-Caffeoyl-4-O-p-Coumaroylquinic acid (Wacyn05614; 8.04) and 1-O-Eudesmoylquinic acid (pmb3062; 7.94), were dramatically enriched in leaves, indicating SA specifically upregulates this compound class. Only four compounds showed root preference, including 3,4-O-Dicaffeoyl-gamma-quinide (PDP158345; −4.30) and 6-O-Dihydrocaffeoylglucose (Zbfn002396; −5.31).

Combined drought and SA treatment (DSAL vs. DSAR) revealed the most extreme tissue-specific partitioning observed (Figure 7D; Supplementary Table S9). Leaf-preferential compounds reached log_2_FC values exceeding 11 [p-Hydroxyphenyl 6-O-(E)-caffeoyl-β-D-glucopyranoside (Ymjm000146; 11.30), dicaffeoylshikimic acid (Lmgp003989; 11.23), trilobatin D (Safn005205; 11.18)], substantially higher than under individual stresses. Numerous caffeoyl conjugates, including 3-O-Caffeoyl-4-O-p-Coumaroylquinic acid (Wacyn05614; 9.66) and ferulate (mws0014; 8.60), reached their highest levels under combined treatment, while only 4-trans-caffeoylglucaric acid (PDP150410; −4.19) remained root-preferential.

Collectively, these results establish that phenolic metabolism in *Ilex rotunda* exhibits strong constitutive tissue-specific partitioning, with leaves serving as the primary hubs for phenolic diversification. Drought stress amplifies this inherent organization, particularly enriching caffeoyl conjugates and galloylated derivatives implicated in foliar protection. SA treatment specifically upregulates caffeoylquinic acid derivatives and dicaffeoyl conjugates, compound classes with documented roles in defense and antioxidant capacity. Critically, combined drought and SA treatment synergistically amplifies both drought- and SA-responsive compound classes, correlating with mitigated stress phenotypes. These findings identify candidate indicators for stress-specific metabolic reprogramming and suggest that exogenous SA applications during moderate drought could simultaneously enhance stress tolerance and boost yields of pharmaceutically valuable phenolics in *Ilex rotunda*.

## 4. Discussion

This study indicates that exogenous SA alleviates drought-induced oxidative damage in *Ilex rotunda* through a strongly tissue-specific reprogramming of phenolic metabolism. Across all datasets, leaves and roots behaved as distinct metabolic compartments, underscoring the importance of root-specific traits in whole-plant stress resilience, with leaves functioning as primary hubs for phenolic diversification and stress-induced reprogramming, whereas roots displayed more constrained but diagnostically informative shifts. By integrating physiological traits, ROS metrics, antioxidant capacity and multivariate metabolomics, we show that SA does not simply add to drought responses but reshapes phenolic networks in a coordinated, tissue-dependent manner.

Drought alone imposed severe oxidative stress, with large increases in MDA, H_2_O_2_ and O_2_·^−^ in both leaves and roots, together with marked chlorophyll loss (Figure 2A–D), consistent with canonical drought-induced photooxidative damage in woody species [48]. However, the accompanying phenolic responses were strikingly divergent between tissues. In leaves, drought increased total phenolics and enhanced DPPH and hydroxyl radical scavenging (Figure 1C,D; Figure 2E,F), linked to upregulation of caffeoyl conjugates and galloyl derivatives (Figure 6A), suggesting reinforcement of the foliar antioxidant barrier. In roots, however, total phenolics and DPPH scavenging declined despite elevated ROS (Figure 1C,D; Figure 2D,E). This was accompanied by strong downregulation of 3,5-dicaffeoylquinic acid (log_2_FC = −7.21; Figure 6D) and enrichment of simpler compounds such as vanillate and 6-O-p-coumaroyl-β-D-glucose, indicating that root phenolic pools are governed by distinct regulatory priorities, possibly reflecting trade-offs between growth, water foraging, and defense in the rhizosphere [49,50]. The temporal hierarchy of drought perception may further explain this divergence: roots encounter water deficit first and bear the brunt of early oxidative stress (3.8-fold increase in superoxide vs. 3.7-fold in leaves; Figure 2D), likely depleting phenolic antioxidants at the primary site of stress, whereas leaves, as secondary sites, retain or accumulate phenolic reserves.

The differential behavior of caffeoylquinic acids across tissues reflects metabolic compartmentalization and divergent selective pressures. Under combined drought and SA, leaves strongly accumulated 1-caffeoylquinic acid (log_2_FC = 6.38) and dicaffeoylshikimic acid (log_2_FC = 10.66), consistent with the role of hydroxycinnamates as antioxidants and UV protectants [8]. Roots markedly suppressed 3,5-dicaffeoylquinic acid while accumulating vanillate. This pattern aligns with the concept that leaves prioritise complex phenylpropanoid conjugates for rapid ROS quenching [48], whereas roots direct carbon toward lignification or rhizosphere exudation rather than caffeoylquinic acid pools species [49,50]. These differences likely reflect continuous photooxidative stress in leaves versus a premium on water uptake and structural integrity in roots. The pronounced root suppression of 3,5-dicaffeoylquinic acid under drought may further represent an adaptive energy-conservation strategy: caffeoylquinic acids are biosynthetically costly [25], and under impaired carbon fixation, down-regulating these complex conjugates while accumulating simpler compounds such as vanillate (log_2_FC = 5.77) could preserve resources for osmotic adjustment, water uptake, and hydraulic maintenance [51]. Partial restoration of 3,5-dicaffeoylquinic acid by SA co-treatment (DSA) suggests that SA alleviates this energetic constraint, possibly by improving plant water status or carbon supply to roots.

SA applied alone elicited a priming-like response. It slightly elevated oxidative markers without compromising chlorophyll content or causing visible damage. Such mild ROS elevations have been proposed as secondary messengers in SA-induced priming of antioxidant systems [22], but they entail a physiological trade-off: maintaining heightened redox sensing and phenolic biosynthesis incurs an energetic cost that could divert resources from growth. In *I. rotunda*, however, SA-treated plants showed no growth penalties or chlorophyll loss, indicating that the primed state is metabolically inexpensive relative to the drought tolerance gained. This aligns with the growth–defense trade-off framework [49,50], where moderate priming optimizes resilience without compromising fitness, especially in long-lived perennials exposed to recurrent stress. Metabolically, SA alone caused broad yet selective remodeling of phenolic profiles. In leaves, it up-regulated caffeoylquinic acids, dicaffeoylshikimic acid, and diverse galloyl conjugates while repressing other caffeoyl-glucose and quinic acid derivatives, suggesting channeling of metabolic flux toward branches with high antioxidant potential. In roots, SA alone largely preserved total phenolic content and clustered metabolically with control roots in HCA and OPLS-DA, indicating that root phenolic networks are comparatively insensitive to SA in the absence of stress. Thus, SA establishes a leaf-centred primed state through targeted enrichment of phenolic antioxidants, consistent with reports in woody perennials where exogenous SA enhances phenylpropanoid metabolism and stress readiness without overt growth penalties.

The combined DSA treatment revealed non-additive rather than purely additive interactions, mirroring findings where SA coordinates stress-induced secondary metabolism across plant families [8]. DSA plants maintained higher chlorophyll levels than drought-stressed plants and showed substantial reductions in MDA, H_2_O_2_, and O_2_·^−^ in both tissues (Figure 2B–D). Antioxidant capacity was concomitantly enhanced: leaf DPPH and hydroxyl radical scavenging remained high, while in roots DSA not only reversed the drought-induced decline in DPPH scavenging but produced the highest hydroxyl radical scavenging rates among all treatments. These physiological benefits were underpinned by distinctive metabolic signatures. In leaves, DSA maintained or increased key SA-responsive metabolites (e.g., 6-O-galloyl-β-D-glucose, 3,6-di-O-caffeoyl-glucose, caffeoylquinic acids) while preserving drought-driven suppression of certain caffeoyl-glucose conjugates. In roots, DSA strongly induced 3,5-dicaffeoylquinic acid and sulfated benzoates—compounds minimally expressed in leaves and strongly downregulated by drought alone. Cross-tissue comparisons under DSA revealed the most extreme leaf–root partitioning observed, with log_2_FC values for several leaf-enriched caffeoyl and galloyl conjugates exceeding those under individual stresses. OPLS-DA and HCA clearly separated DSA leaves and roots from all other groups, and permutation tests confirmed robustness. Collectively, SA and drought co-activate complementary phenolic branches in a tissue-specific manner: SA amplifies leaf-localised caffeoylquinic and galloyl pathways supporting high antioxidant capacity, while in roots it restores or de novo induces drought-sensitive phenolic classes, thereby re-establishing belowground antioxidant competence. PCA revealed that DSAR clustered with DR, whereas DSAL diverged from DL (Figure 4D), confirming stronger metabolic reprogramming in leaves than roots under drought.

The strong tissue specificity observed here aligns with emerging concepts of organ-level metabolic specialisation in woody plants [49]. Even under control conditions, leaves harbored a much richer phenolic repertoire, with 44 of the top 50 differentially accumulated metabolites being leaf-enriched; drought and SA amplified this inherent organisation by boosting complex caffeoyl and galloyl polymers. These compounds are potent ROS scavengers and UV screens and may contribute to cell wall reinforcement and pathogen resistance [52,53]. Roots maintained a smaller but distinct phenolic set, including 2-hydroxyphenylacetate and 5-hydroxyferulic acid, whose levels remained relatively stable across treatments and may be linked to rhizosphere interactions or lignification resistance [51]. The persistence of this root-preferential core, even under severe drought and DSA, suggests tight buffering of certain root phenolic functions against environmental perturbation.

Our results are broadly consistent with previous work showing that exogenous SA enhances drought tolerance in woody species by modulating antioxidant systems and secondary metabolism [32]. For example, SA priming promotes sorghum germination under drought through metabolic reprogramming [54], alleviates combined cadmium and drought stress in *Pterocarya fraxinifolia* by regulating water status and antioxidant defence [55], and engages in crosstalk with ABA and the phenylpropanoid pathway in soybean seeds [56]. The present study extends these findings by providing a high-resolution, tissue-resolved metabolomic view in a medicinal tree, revealing that SA–drought synergy is underpinned by coordinated, organ-specific reallocation of phenolic flux rather than uniform up-regulation. This has practical implications: targeted SA applications during moderate drought may not only stabilise photosynthetic performance but also enhance the yield of pharmaceutically valuable phenolics, particularly caffeoylquinic acids and galloyl derivatives, in *I. rotunda* foliage.

Several limitations should be acknowledged. Experiments were conducted under controlled conditions at a single sampling time, precluding assessment of temporal dynamics and field-level variability. While the bark is the traditional medicinal material, it was not sampled; future work should examine whether SA-induced foliar phenolic shifts translate to bark metabolite profiles, given the logistical and sustainability challenges of bark harvesting. Although our metabolomic and physiological data reveal strong associations between specific phenolic reprogramming and reduced oxidative markers, the study remains correlative; establishing causation will require enzyme inhibition, pathway-specific gene expression analyses, or targeted metabolite feeding/knockout studies to validate the functional roles of the candidate indicators identified here. The regulatory mechanisms underlying tissue-specific phenolic reprogramming remain unresolved, as gene expression and signaling components were not examined. Although chloroplast and mitochondrial genomes of *I. rotunda* have been characterized [57,58], the lack of a nuclear genome reference and validated internal control genes (e.g., Actin) for qPCR constrained targeted gene expression analysis. Future studies integrating time-series metabolomics, transcriptomics (e.g., RNA-seq), enzyme activity assays, and osmolyte profiling (proline, soluble sugars) would collectively dissect the molecular control of SA-responsive pathways, identify key transcription factors [59], and elucidate the cooperative interplay between osmotic adjustment and ROS scavenging in SA-mediated drought tolerance. The recent publication of chromosome-scale genomes for related Ilex species [60,61] may serve as valuable references. Additionally, field trials are needed to evaluate long-term SA application strategies, optimal dosing, and potential tradeoffs between growth, stress tolerance, and metabolite production in *I. rotunda* plantations.

## 5. Conclusions

We hypothesized that exogenous SA alleviates drought-induced oxidative damage by enhancing phenolic-based antioxidant capacity through distinct yet coordinated metabolic adjustments in leaves and roots. Our findings confirm this hypothesis: SA priming pre-emptively enriched protective phenolic classes without growth penalties, and combined drought + SA treatment elicited synergistic tissue-specific responses that exceeded additive effects. In leaves, this synergy amplified caffeoylquinic and galloyl conjugates, markedly lowering oxidative markers (MDA reduced by 44%), whereas in roots SA partially reversed the drought-induced suppression of costly metabolites like 3,5-dicaffeoylquinic acid, restoring antioxidant competence while conserving carbon. These coordinated, organ-specific metabolic shifts directly validate that SA-mediated protection operates through differential network regulation rather than passive stress superposition. The identified phenolic biomarkers provide a mechanistic basis for targeted resilience strategies in woody perennials, although the molecular regulators of this tissue-specific synergy remain to be elucidated under field conditions.

## Data Availability

All data supporting the findings of this study are available within the article and its Supplementary Materials (Tables S1–S9). Raw metabolomics data are provided as peak areas and fold-change values in the supplementary tables. Further inquiries can be directed to the corresponding authors.

## Supplementary Materials

The following supporting information can be downloaded at https://www.mdpi.com/article/10.3390/antiox15070808/s1: Table S1: Phenolic compounds with confidence levels, ionization mode, Q1, Q3, and chemical formula of identified metabolites. Table S2: DL vs. CKL differentially altered phenolic compounds. Table S3: SAL vs. CKL differentially altered phenolic compounds. Table S4: DSAL vs. CKL differentially altered phenolic compounds. Table S5: DR vs. CKR differentially altered phenolic compounds. Table S6: CKL vs. CKR differentially altered phenolic compounds. Table S7: DL vs. DR differentially altered phenolic compounds. Table S8: SAL vs. SAR differentially altered phenolic compounds. Table S9: DSAL vs. DSAR differentially altered phenolic compounds.
